# Small Pox

**Published:** 1829

**Authors:** 


### III. ORIGINAL.

43. *Small Pox.*—During the past winter the small pox prevailed to some extent in Pittsburgh, whence cases of it were introduced into several towns on the Ohio below, but as far as we have learned, it did not spread in any of them. Nine persons infected with it, reached Cincinnati at different times, and were all sent to a house provided for their reception in the vicinity of the city. No one contracted the disease from them. The arrival of these individuals excited some salutary alarm, and led to a revival of the practice of vaccination. Physicians were appointed by the Council to vaccinate all the unvaccinated in the different wards of the city, and were paid out of the public treasury, for attending on those who were unable to make compensation. This was right, and we hope the Council will consider it their duty to do the same thing annually. We also hope, that they will repeal the ordinance providing for the removal of infected persons from the city, and make it publicly known, that henceforward the people must depend on vaccination, alone, to protect them against the contagion of small pox. By such a regulation, coupled with appropriations for the benefit of the poor, we might hope to keep the number of liable persons so small, as effectually to preclude the small pox from becoming epidemic.

It was not without concern, that, while the subject was agitated, we found so many persons unsettled in their opinions respecting the preventive power of the vaccine disease. We have even been told, that there are physicians in the Western country, who inculcate doubts on this subject. That the *people* should want perfect confidence is not remarkable, seeing that most of them have had no opportunities of witnessing the efficacy of the vaccine in preventing the variolous disease; and that most of them are ignorant of the historical evidences of its virtue. The profession must inform them.

As domestic proofs are always more convincing than foreign, we take this opportunity of saying, that during the winter of 1828—9, and two years before, when a still greater number of cases of small pox were introduced here, no person (as far as we know) who had been vaccinated, contracted the disease, although many such were exposed. In both instances the nurses and physicians in daily attendance, confided entirely in their previous vaccination, and escaped the disease. The Editor of this Journal, in the discharge of his duty as President of the Board of Health, was several times exposed with impunity, although more than a quarter of a century had elapsed since he was vaccinated. Other medical gentlemen, belonging to the same Board, were likewise exposed and proved to be equally unsusceptible. One of the cases which lately occurred, was peculiarly interesting in reference to this question. Two young mechanics, brothers and natives of New England, on their way to Cincinnati lodged one night in Pittsburgh, where the small pox was epidemic. Soon after reaching this city, one of them was taken down with that malady, and sent to the small pox house, where there were other patients at the same time. The brother attended him as a nurse, lodged on the same bed with him, and performed every kind of personal services till he died and was buried. Now the deceased had never been vaccinated, but the surviving brother had, and to this only can we ascribe the difference in their fate.

